# The Experience of a Tertiary Reference Hospital in the Study of Rare Neurological Diseases

**DOI:** 10.3390/medicina59020266

**Published:** 2023-01-30

**Authors:** Styliani-Aggeliki Sintila, Marina Boziki, Christos Bakirtzis, Thomai Stardeli, Nikoletta Smyrni, Ioannis Nikolaidis, Dimitrios Parissis, Theodora Afrantou, Theodore Karapanayiotides, Ioanna Koutroulou, Virginia Giantzi, Paschalis Theotokis, Evangelia Kesidou, Georgia Xiromerisiou, Efthimios Dardiotis, Panagiotis Ioannidis, Nikolaos Grigoriadis

**Affiliations:** 1 2nd Department of Neurology, AHEPA University Hospital, School of Medicine, Faculty of Health Sciences, Aristotle University of Thessaloniki, 54636 Thessaloniki, Greece; 2 Laboratory of Experimental Neurology and Neuroimmunology, 2nd Department of Neurology, AHEPA University Hospital, 54636 Thessaloniki, Greece; 3 Department of Neurology, University Hospital of Larissa, Faculty of Medicine, School of Health Sciences, University of Thessaly, 41110 Larissa, Greece

**Keywords:** rare neurological diseases, reference center, orphan disease, neurodegenerative disease, clinical genetics

## Abstract

*Background and Objectives:* Rare diseases (RDs) are life-threatening or chronically impairing conditions that affect about 6% of the world’s population. RDs are often called ‘orphan’ diseases, since people suffering from them attract little support from national health systems. *Aim:* The aim of this study is to describe the clinical characteristics of, and the available laboratory examinations for, patients who were hospitalized in a tertiary referral center and finally received a diagnosis associated with a Rare Neurological Disease (RND). *Materials and Methods:* Patients that were hospitalized in our clinic from 1 January 2014 to 31 March 2022 and were finally diagnosed with an RND were consecutively included. The RND classification was performed according to the ORPHAcode system. *Results:* A total of 342 out of 11.850 (2.9%) adult patients admitted to our department during this period received a diagnosis associated with an RND. The most common diagnosis (N = 80, 23%) involved an RND presenting with dementia, followed by a motor neuron disease spectrum disorder (N = 64, 18.7%). Family history indicative of an RND was present in only 21 patients (6.1%). Fifty-five (16%) people had previously been misdiagnosed with another neurological condition. The mean time delay between disease onset and diagnosis was 4.24 ± 0.41 years. *Conclusions:* Our data indicate that a broad spectrum of RNDs may reach a tertiary Neurological Center after a significant delay. Moreover, our data underline the need for a network of reference centers, both at a national and international level, expected to support research on the diagnosis and treatment of RND.

## 1. Introduction

Rare diseases (RDs) are life-threatening or chronically impairing conditions that affect about 6% of the world’s population [1]. RDs are often called ‘orphan’ diseases, since people suffering from them attract little support from national health systems. In addition, research concerning their pathogenesis and treatment is scarce, mainly due to limited funding opportunities [2]. ‘Orphan drug’ development is of uncertain profit; therefore, it is limited [3].

Due to the unusual presentation, insufficient diagnostic tools and lack of physicians’ expertise, the diagnosis of RDs is often delayed or missed. After diagnosis, prognosis and treatment of RDs is also challenging. Since the number of people with RDs is relatively small and for many years patients are misdiagnosed, the wide cooperation of researchers and treating physicians is strongly recommended by international health committees. One such initiative is the Orphanet database which serves as a source of information on RDs and provides users with access to expert services around the globe. More than 40 countries are currently supporting Orphanet database, using the ORPHAcode system in order to describe and classify RDs [4]. In Europe, the European Reference Network [5] and the ITHACA network (on Rare Congenital Malformations and Rare Intellectual Disability) [6] support the cooperation of researchers and healthcare providers in this field, while a network specifically for Rare Neurological Diseases (RNDs) has been developed in the frame of the ERN [7].

More than 8000 rare diseases have been described and affect between 6% and 8% of the European population, with significant prevalence variability across countries [8]. The majority (80%) of RDs is of a genetic origin and predominantly affects children [9]. A small number of them is attributed to environmental exposures during pregnancy or later in life, often combined with genetic vulnerability. The remaining are rare cancers, auto-immune diseases, congenital malformations and toxic and infectious diseases [10], and only a small proportion is considered treatable.

According to the Physicians ‘Guide to Rare Diseases [11], more than half of the people with RDs present symptoms that require neurological consultation. Regarding RNDs, the most frequent conditions, as described in the Italian initiative for RDs [12], are amyotrophic lateral sclerosis (ALS), muscular dystrophies, spinocerebellar ataxias, hereditary neuropathies, myotonic dystrophy and Huntington’s disease. A wide range of diseases being classified as neurodegenerative are considered as very rare.

The aim of this study was to describe the clinical characteristics and the available laboratory examinations that were applied for the diagnostic workup of patients who were hospitalized in a tertiary referral center and finally received an RND diagnosis in an 8-year time frame. In addition, we focused on the possible time delay between the onset of symptoms and the diagnosis.

## 2. Materials and Methods

### 2.1. Database Description

This is a retrospective analysis conducted on a digital database that included all adult patients who were admitted in the 2nd Department of Neurology, AHEPA University Hospital of Thessaloniki, from 1 January 2014 to 31 March 2022 and received a diagnosis associated with an RND. The RND classification was performed according to the ORPHAcode system. The analyzed data included family history and diagnostic tools that enabled the diagnosis, such as brain magnetic resonance imaging (MRI), nerve conduction study (NCS), electromyography (EMG), electroencephalography (EEG), single-photon emission computed tomography (SPECT), positron emission tomography (PET), dopamine transporter scan (DAT-Scan), tissue biopsy and other laboratory and genetic testing. We also included the time lag between onset of symptoms and disease diagnosis, as well as previous diagnosis status. The study received the approval of the Bioethics’ Committee of the School of Medicine of the Aristotle University of Thessaloniki (Approval Nr. 4592/21 February 2022). All participants signed an informed consent. This is a retrospective analysis of data; the informed consent of the participants was supervised by the Bioethics’ Committee according to the National Registration of personal data.

### 2.2. Statistical Analysis

The statistical analysis was performed using SPSS Version 25.0 (Armonk, NY, USA: IBM Corp.). A descriptive analysis was performed by calculating the frequency for qualitative variables (number of patients with a specific type of neurological disease, number of diagnostic procedures and number of patients with a previous correct/incorrect diagnosis) and the mean time from disease onset to disease diagnosis across disease categories.

## 3. Results

A total of 342 out of 11.850 (2.9%) hospitalized adult patients in our clinic from 1 January 2014 to 31 March 2022, were finally diagnosed with an RND. A slight predominance of the male patients was observed (n = 176, 51.5%) and the mean age at diagnosis was 56.5 ± 0.9 years. It is worth mentioning that the mean age at diagnosis for patients with dementia due to a neurodegenerative disease was 66.5 ± 0.8 years, whereas patients with rare epilepsies were diagnosed, on average, 42 years earlier (mean age = 24 ± 3 years). This difference is highly related to the clinical characteristics of epilepsies as well as the slow clinical course and the mild to moderate intensity of neurodegenerative diseases as time goes by.

**Figure 1:**
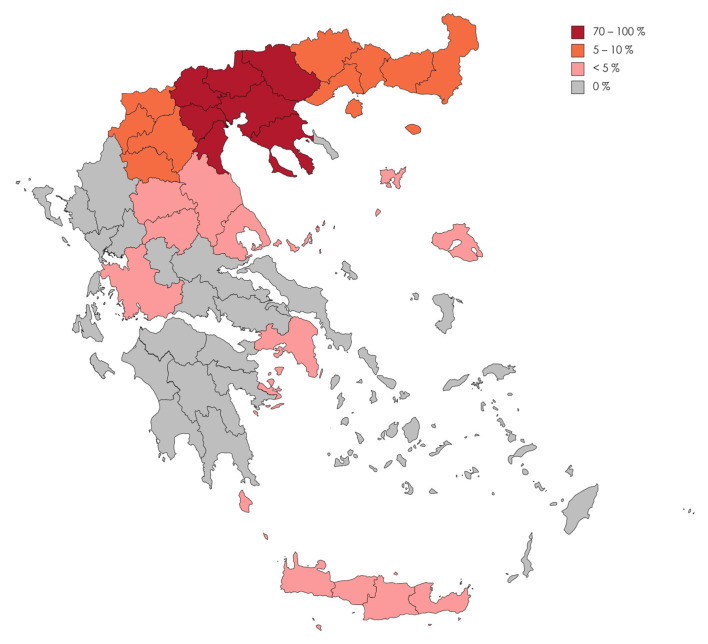
A map of Greece and the frequency of Rare Neurological Diseases admissions to the 2nd Department of Neurology, AHEPA University Hospital of Thessaloniki, according to the geographic regions of Greece.

The spectrum of diagnosed RNDs is categorized in Table 1. The RNDs diagnosed are further classified by their disease category and are presented as Supplementary Material (Table S1). The most common diagnoses (n = 80, 23%,) involved an RND presenting with dementia, followed by the motor neuron disease spectrum (n = 64, 18.7%). The majority of these patients (78.6%) lived in Central Macedonia and only a minority in Thessaly (1.8%), Western Greece (0.9%), Attika (0.9%), Crete (0.3%) and the North Aegean islands (0.3%; Figure 1). One patient lived in the United Kingdom, one in Sofia, Bulgaria, and another one in Ostrava, Czech Republic. Our center, based in Thessaloniki, in Central Macedonia, Greece, accounts for these results to a large extent.

**Figure 2:**
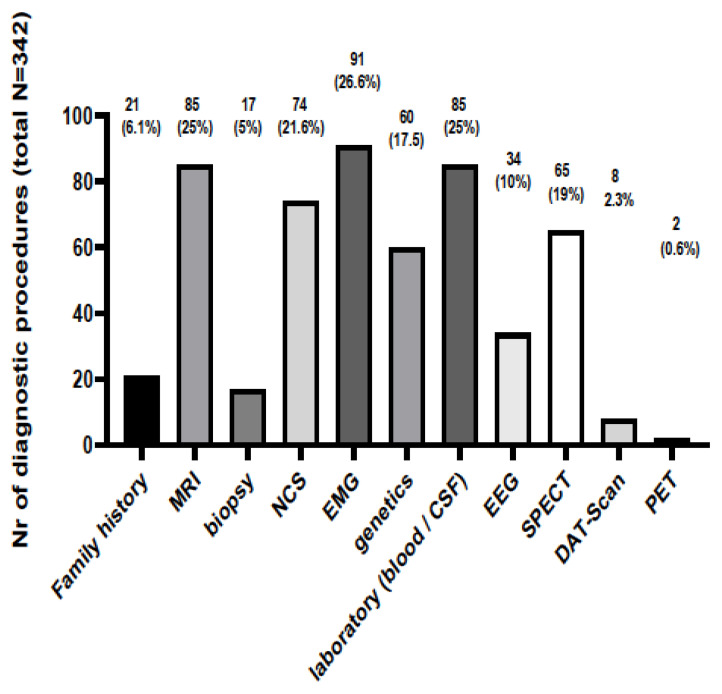
The number of diagnostic procedures and the clinical data that led to the diagnosis of a Rare Neurological Disease. MRI: Magnetic Resonance Imaging; NCS: Nerve conduction study; EMG: Electromyography; CSF: Cerebrospinal fluid; EEG: Electroencephalography; SPECT: Single-photon emission computed tomography; DAT-Scan: Dopamine Transporter Scan; PET: Positron Emission Tomography.

The diagnostic procedures and clinical data that led to the diagnoses are presented in Figure 2. Standard diagnostic tools and procedures such as MRI, EEG, EMG, NCS and family history were implicated in most cases (305/342, 89.2%) in order to support the diagnosis. Electrodiagnostic examinations were commonly used in order to diagnose an RND involving the peripheral nervous system and muscles; EMG guided the diagnosis in 91 (26.6%) people, whereas NCSs were performed in 74 (21.6%) patients. SPECT, brain MRI and EEG were performed in 65 (19%), 85 (25%) and 34 (10%) patients, respectively. Genetic testing was diagnostic in 44 (72.1%) out of a total of 61 patients being tested due to a suspected underlying pathology related to an RD. Table 2 presents the RNDs in which genetic testing was carried out, the genetic results and the confirmation rates (for each disease and as a whole). The types of genetic tests performed to reach the diagnosis with certainty are presented at Table 3. In the majority of cases, a single-gene targeted analysis was performed. A whole-exome sequence was performed to diagnose Argininemia and Hereditary diffuse leukoencephalopathy with spheroids; whereas, in cases of Kearns Sayre and MELAS, mitochondrial DNA was sequenced and analyzed.

The observed low frequency of genetic testing performed is mainly attributed to the cost of the examination, and the limited number of genetic polymorphisms linked with an RND available for testing via public insurance. More extended genetic testing might have yielded a higher confirmation rate via genetic testing and, in addition, its implementation would avoid a series of other costly procedures. Nevertheless, among the 61 patients eligible for genetic testing who underwent the procedure, a correct diagnosis was confirmed in more than half of them (n = 44, 72.1%). This result highlights the importance of increased clinical suspicion that might guide the referral for genetic testing.

Laboratory examinations and biopsies were crucial in establishing the diagnosis in 85 (25%) and 17 (5%) of cases, respectively. Among the laboratory examinations, biochemistry tests were performed. Serum was analyzed for inflammatory markers and Abs associated with paraneoplastic, autoimmune, rheumatologic, neuromuscular disorders and peripheral neuropathies. Blood was also studied for lysosomal enzyme activity (dried blood spot for enzymatic analysis to exclude or confirm leukodystrophies). CSF analysis was performed as well (pressure, protein, cell count, IgG index, oligoclonal bands, cytology, 14-3-3 protein, RT-QuIC test, PCR for JCV DNA, tau and beta-amyloid as biomarkers for Alzheimer disease, anti-GAD and anti-NMDAR Abs titer) as well as urine analysis (urinary 24-h copper excretion to exclude Wilson disease). Polysomnography was performed to diagnose narcolepsy.

**Figure 3:**
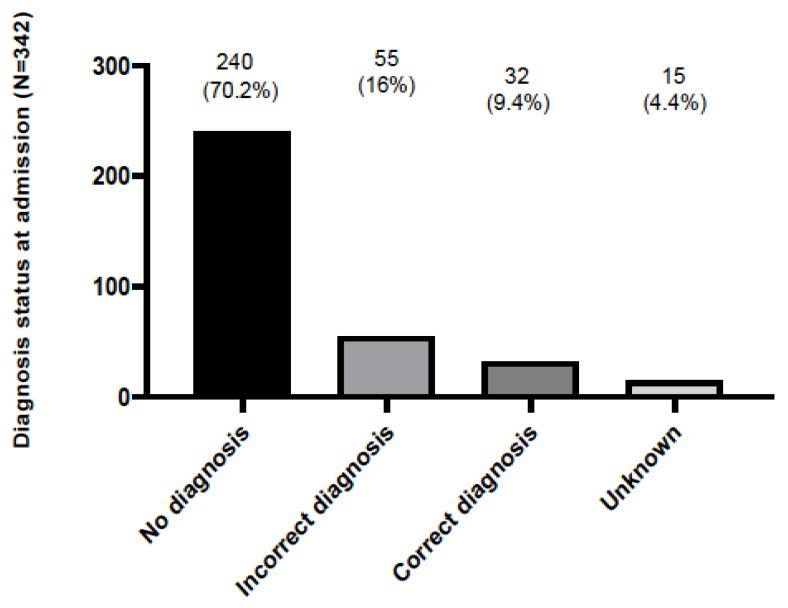
The frequency of patient admissions to the 2nd Department of Neurology, AHEPA University Hospital of Thessaloniki and the resulting diagnosis: patients were either undiagnosed (no diagnosis), had received a wrong diagnosis (incorrect diagnosis) or were previously diagnosed correctly (correct diagnosis). Unknown means lacking data.

A positive family history indicative of an RD was present only in 21 patients (6.1%), possibly indicating that the significance of symptoms in previous generations was underestimated by patients and family members. It could also be attributed to incomplete disease penetrance across generations with older patients manifesting milder disease phenotypes [13]. Interestingly enough, 55 (16%) and 15 (4.4%) out of 342 patients had either been previously misdiagnosed, or received an inconclusive diagnosis, respectively (Figure 3). Overall, the mean delay between the onset of symptoms and disease diagnosis was 4.24 ± 0.41 years. For each RND group, the mean diagnostic delay (in years) was 3.33 ± 0.44 for the neurodegenerative diseases, 3.86 ± 0.66 for the neuromuscular diseases, 3.05 ± 1.2 for the neuroinflammatory–immunological diseases, 5.66 ± 3.7 for the rare ataxias, 4.5 ± 2.4 for the rare vascular diseases, 8.2 ± 2.6 for the rare peripheral neuropathies, 14.5 ± 6.4 for the neurocutaneous syndromes; no delay was identified for the central nervous system malformation cases.

Regarding the therapeutic interventions after a confirmed diagnosis, only 50 (14.6%) out of 342 patients received an orphan drug. Among them, two patients (0.6%) with spinal muscular atrophy type III received nusinersen, an antisense oligonucleotide. Another two patients (0.6%) received patisiran, a transthyretin (TTR)-directed double-stranded, small interfering RNA, for the treatment of the polyneuropathy of hereditary TTR-mediated amyloidosis (hATTR). One patient (0.3%) was treated with long-term enzyme replacement therapy for Pompe disease (alpha-glucosidase) and another one (0.3%) received brivaracetam and metformin for Lafora body disease, a type of rare, inherited and severe progressive myoclonic epilepsy. Forty-four patients (13%) suffering from amyotrophic lateral sclerosis received riluzole either as tablet or as oral suspension. Four out of 342 patients (1.1%) were lost to follow-up.

**Figure 4:**
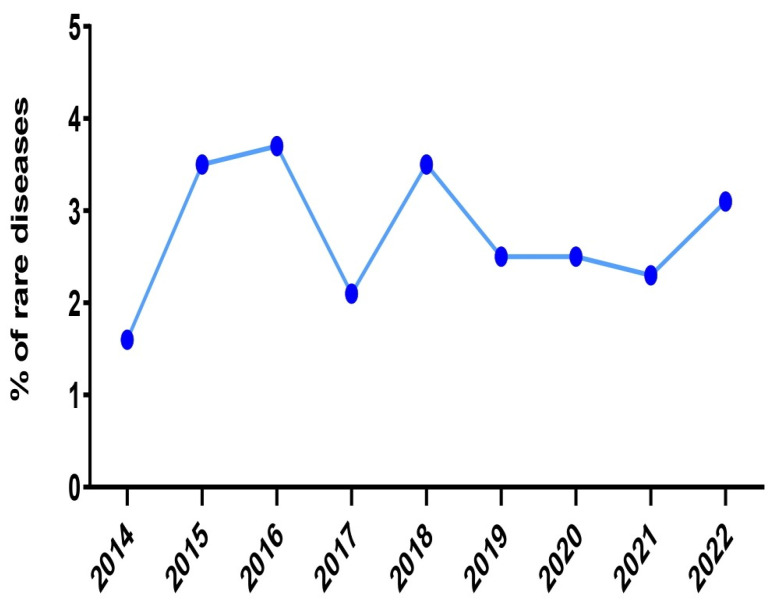
The incidence of rare diseases in the 2nd Department of Neurology, AHEPA University Hospital of Thessaloniki over the years 2014, 2015, 2016, 2017, 2018, 2019, 2020, 2021 and the first trimester of 2022.

Concerning the incidence of RND cases that were admitted in our department, the rates displayed a remarkable decline during the biennium 2020–2021 (Figure 4). In particular, during the year 2020, among the 1036 patients that were hospitalized, twenty-seven patients (2.6%) were diagnosed with an RND. This percentage was even lower the next year (2.3%), although the absolute number of newly diagnosed RND cases was almost stable (28 out of 1200 hospitalized patients). In contrast, the highest rates were recorded during the decade 2015–2016 and the year 2018 (3.5%, 3.7% and 3.5%, respectively). The decline in the incidence coincides temporally with the COVID-19 pandemic and this could have significantly affected our results.

## 4. Discussion

In the context of the present study, the RNDs most often diagnosed in adults were neurodegenerative diseases accompanied by dementia, followed by rare motor neuron disorders. Standard diagnostic tools and procedures were used in the majority of cases in order to obtain a diagnosis. Of note, many RNDs may be diagnosed by the use of standard equipment in neurologic clinics, as long as physicians are familiar with these disease entities. This fact highlights the need for a sufficient number of physicians to train towards the clinical suspicion of RNDs in order to implement timely screening procedures and/or to refer patients to specialized departments and, if possible, to RND Centers of Excellence. It is necessary to implement educative training originating from RND reference centers tailored for neurologists and/or other medical professionals that are likely to manage RND-related symptoms [8].

Although the majority of our patients lived in Thessaloniki, in the premises of Thessaloniki and/or Central Macedonia, 3.9% of them lived in Attica, Thessaly, Crete, Western Greece and North Aegean and 0.9% lived abroad. These data highlight that the highest priority is collaboration among specialized neurological centers and primary care units at a national and international level. A comprehensive approach, in terms of investigation and diagnosis of RNDs dictates that there are no geographical limits during the journey of a patient with a rare disease until his final diagnosis.

The mean time from the first symptom to the final diagnosis was approximately four years. Moreover, a significant number of patients initially received a mistaken diagnosis. Our results confirm previous evidence indicating that the correct diagnosis of RNDs is often delayed due to an unusual, insidious clinical presentation and the lack of the clinicians’ relevant knowledge [2]. In our study this was particularly evident in relation to neurocutaneous diseases, the phenotypically and genetically discrete multisystem disorders that essentially affect the skin and the CNS. Their diagnosis is mainly clinical; however, genetic testing is necessary in unusual cases. Moreover, clinical features often manifest gradually, thus making their diagnosis at a young age rather difficult [14].

A small number of RNDs are treatable, especially in the early stages, thus allowing relief from symptoms and an improved quality of life [15]. However, access to orphan drugs requires consultation from highly specialized RND Centers. Therefore, the prompt referral by clinicians to national networks of RD reference centers is expected to enable earlier diagnosis, effective management and, if possible, timely treatment.

In the present patient cohort, 12.8% of diagnoses (44 out of 342) were established by the use of genetic testing. Several advances have taken place in the field of clinical genetics the last ten years and these advances have accelerated diagnosis for many RNDs. Access to Next Generation Sequencing techniques, such as whole exome sequencing should be facilitated for cases with the suspected genetic etiology, where necessary [16,17].

In our experience, access to the essential genetic testing procedures, where necessary, is crucial for the management of degenerative dementias [18,19,20,21] and other rare diseases such as neuromuscular [22,23], inflammatory and autoimmune [24,25,26,27,28,29], peripheral neuropathies [24,30] and cerebrovascular rare diseases [31].

Another noteworthy observation arising from our study is the impact of the COVID-19 pandemic on the hospitalization of patients with neurologic rare diseases. Notably, RND admissions to our clinic were consistently reduced during the second wave of the pandemic. The experience from a referral center for dermatologic rare diseases in Southern Italy recorded a significant reduction in the number of admissions at their clinic in the year 2020, but not in 2021, attributing this outcome to the implementation of strict, protective measures against COVID-19 infection [32]. Another (cross-sectional) study on 170 patients suffering from 89 different rare diseases in Hong Kong also examined the impact of the COVID-19 pandemic. The results demonstrated that the COVID-19 infection influenced patients’ health status as well as their access to the medical and rehabilitation services [33]. Moreover, a COVID-19 infection can significantly alter the course or accelerate the onset of some symptoms if the infection involves people with neurological rare diseases. Similar results regarding the limited access to health care arose from a multi-country survey that studied the sequalae of coronavirus on a rare disease European community [34].

Presumably, the restrictions that the coronavirus disease dictated, especially social isolation and complete lockdown, might have had a negative impact on the behaviors of RND patients. Rare diseases are chronic conditions that could make patients more vulnerable to the stress-related harmful effects of the COVID-19 pandemic [35]. The feelings of anxiety and insecurity regarding the effective prevention of the SARS-CoV-2 infection could justify the restricted access to the public healthcare system, and hence our clinic.

This is the first epidemiological retrospective study in the Greek population over the course of an 8-year time interval. The limitations of this study include the relatively small number of patients in some subgroups, the fact that this is a single center study and the lack of comparative data from people with an RD of other specialties that were admitted in this hospital during this study period.

## 5. Conclusions

Our data indicate that, following symptom onset, patients with a broad spectrum of RNDs may reach a tertiary neurological center after a significant delay frequently approximating 4 years. Notably, concrete evidence of a family history may be lacking, especially for patients with symptom onset in the adulthood, thus posing a challenge for clinical suspicion to arise. Current advances in diagnostic algorithms have enabled the discovery and identification of RNDs. However, limited expertise may prevent early detection. Moreover, access to genetic testing is often subjected in cost-related limitations, especially for cases in need of advanced sequencing investigation techniques.

Our data underline the need for a network of reference centers for RNDs, at both a national and an international level, which are expected to support research on RND diagnosis and treatment. These centers may orchestrate the training of physicians also in collaboration with primary care facilities, considering that a number of RNDs may be suspected and investigated, at least in part, by the use of standard diagnostic tools. Physicians are encouraged to further investigate the signs and symptoms that could lead to an RND diagnosis. The training material provided by the initiatives mentioned above could be a valuable resource for information on RNDs. The heterogeneity, complex presentation and distinct manifestations pose difficulties with the management of rare disease patients. These patients need more medical attention than the general population, with or without pandemics.

## Figures and Tables

**Table 1:** Rare Neurological Diseases diagnosed and relevant ORPHAcode classification.

Neurodegenerative diseases with dementia	98534	80	23
Rare motor neuron diseases	98503	64	18.7
Rare peripheral neuropathies	98496	30	8.8
Rare diseases of the neuromuscular junction	98491	28	8.2
Rare neuroinflammatory and neuro-immunological disease	182064	19	5.5
Genetic diseases of the skeletal muscle	206634	19	5.5
Neurodegenerative diseases with parkinsonism	306666	11	3.2
Other rare neurodegenerative diseases	182070	10	3
Rare systemic or rheumatologic disease with CNS involvement	98023	10	3
rare movement disorders	102003	10	3
Rare neurodegenerative diseases with chorea	306719	9	2.6
Other rare diseases		9	2.6
Rare vascular diseases of the CNS	71281	8	2.3
Rare ataxias	102002	7	2
Neurocutaneous syndrome with epilepsy	166466	6	1.8
Rare epilepsies	101998	6	1.8
Rare inborn errors of metabolism	68367	4	1.1
Acquired diseases of the skeletal muscle	206638	4	1.1
CNS malformations	98044	2	0.6
Rare headache	98022	2	0.6
Medullar disease	139417	2	0.6
Rare sleep disorder	68354	1	0.3
Rare nervous system tumor	98062	1	0.3

CNS: Central Nervous System; n: number of patients.

**Table 2:** Number of Rare Neurological Diseases in which a genetic test was undertaken; the genetic results and the confirmation rates are also presented.

Diseases	Frequency (n/60)	Genetic Result (Positive, Negative or Unknown)	Genetic Confirmation (%)
Argininemia	1	positive	100
Lafora Disease	1	positive	100
Becker myotonia	3	positive	100
C9orf72 (MND-FTD)	3	unknown = 2, negative = 1	0
CADASIL	2	positive = 1, unknown= 1	50
CMT-1A	4	positive	100
CMT-X	1	positive	100
Congenital myasthenia	1	positive	100
DM1	5	positive	100
DM2	2	positive	100
Dystrophinopathy	1	positive	100
Friedreich ataxia	1	positive	100
FSHD	1	positive	100
hATTR	2	positive	100
Hereditary Alzheimer Disease	1	negative	0
Hereditary diffuse leukoencephalopathy with spheroids	1	positive	100
HNPP	1	positive	100
Huntington’s disease	13	positive = 9, negative = 3, unknown = 1	69
Kearns Sayre	1	positive	100
Leukodystrophy	2	unknown = 1, negative = 1	0
LGMD1C	1	negative	0
MELAS	2	positive	100
neuro-Behcet	1	positive	100
NF1	1	positive	100
Niemann-Pick C	1	negative	0
POMPE disease	1	positive	100
SCA 1,2,3,6,7	2	negative	0
SCA 1,2,3,6,7, Niemann Pick C, and Friedreich ataxia	1	negative	0
SMA	3	positive	100
Thomsen myotonia	1	negative	0
Total	61	POSITIVE = 44, NEGATIVE = 12, UNKNOWN = 5	YES = 72.1 %, NO = 20%, UNKNOWN = 8.3%

CMT-1A: Charcot-Marie-Tooth type 1A; CMT-X: X-linked Charcot-Marie-Tooth disease; DM1: Myotonic dystrophy type 1; DM2: Myotonic dystrophy type 2; MND-FTD: motor neuron disease- frontotemporal dementia variant; MELAS: Mitochondrial encephalomyopathy, lactic acidosis and stroke-like episodes; NF1: Neurofibromatosis type 1; SMA: spinal muscular atrophy; LGMD1C: Limb girdle muscular dystrophy type 1C; HNPP: Hereditary neuropathy with pressure palsies; CADASIL: cerebral autosomal dominant arteriopathy with subcortical infarcts and leukoencephalopathy; hATTR: hereditary transthyretin amyloidosis; SCA 1,2,3,6,7: spinocerebellar ataxias type 1,2,3,6,7; FSHD: Facioscapulohumeral Muscular Dystrophy.

**Table 3:** Type of genetic testing performed to reach the diagnosis with certainty.

DISEASE	Type of Genetic Test
CMT-1A	single-gene analysis (PMP22 duplication)
CMT-X	single-gene analysis (GJB1 gene, protein connexin32)
DM1	single-gene analysis (targeted analysis of the DMPK gene for CTG trinucleotide expansions)
C9orf72 (MND-FTD)	Targeted analysis for G4C2 hexanucleotide expansions in the C9orf72 gene
Hereditary Alzheimer Disease	NGS-based gene panel (negative for APP, PSEN1, PSEN2)
Hereditary diffuse leukoencephalopathy with spheroids	whole-exome sequence (heterozygous point mutation in the CSF1R gene)
Huntington’s disease	targeted analysis of the Huntington gene (HTT gene) for CAG trinucleotide expansions
Kearns Sayre	Deletion/duplication analysis of mtDNA
MELAS	entire mitochondrial genome sequence
Leukodystrophy	single gene tests or small gene panels, Dried Blood Spot for enzymatic analysis
neuro-Behcet	gene-targeted testing (HLA-B*51 sequence analysis)
NF1	sequence analysis of NF1 genomic DNA or complementary DNA
SMA	single-gene analysis (gene-targeted analysis of SMN1 gene on chromosome 5q13)
Friedreich ataxia	single-gene analysis (GAA repeat expansion in intron 1 of frataxine gene)
LGMD1C	NGS-based gene panel (CAV3 gene-LGMD1C)
Dystrophinopathy	single-gene analysis (of DMD)
HNPP	single-gene analysis (PMP22 deletion on chromosome 17)
Congenital myasthenia	gene-targeted testing (multi-gene panel)
DM2	single-gene analysis (targeted analysis of the CNBP gene for CCTG expansion in intron 1)
CADASIL	NGS-sequence variants in the NOTCH3 gene
POMPE disease	gene-targeted analysis and acid alpha-glucosidase (GAA) enzyme activity performed on dried blood spots
hATTR	Single-gene testing (sequence analysis of TTR gene)
Niemann-Pick C	gene-targeted testing (multigene panel that includes NPC1, NPC2)
SCA 1,2,3,6,7	multigene “repeat expansion” panel (targeted variated analysis to specifically identify CAG repeat expansion)
Thomsen and Becker myotonia	Single-gene testing (sequence analysis of CLCN1 gene)
FSHD	single-gene testing (targeted analysis for the repeat size of the D4ZA on chromosome 4q35)
Argininemia	whole-exome sequence (mutations in the ARG1 gene)
Lafora Disease	Single-gene testing (sequence analysis of EPM2A and NHLRC1 genes)

CMT-1A: Charcot-Marie-Tooth type 1A; CMT-X: X-linked Charcot-Marie-Tooth disease; DM1: Myotonic dystrophy type 1; DM2: Myotonic dystrophy type 2; MND-FTD: motor neuron disease- frontotemporal dementia variant; MELAS: Mitochondrial encephalomyopathy, lactic acidosis and stroke-like episodes; NF1: Neurofibromatosis type 1; SMA: spinal muscular atrophy; LGMD1C: Limb girdle muscular dystrophy type 1C; HNPP: Hereditary neuropathy with pressure palsies; CADASIL: cerebral autosomal dominant arteriopathy with subcortical infarcts and leukoencephalopathy; hATTR: hereditary transthyretin amyloidosis; SCA: 1,2,3,6,7: spinocerebellar ataxias type 1,2,3,6,7; FSHD: Facioscapulohumeral Muscular Dystrophy, PMP22: Peripheral Myelin Protein 22; GJB1: Gap Junction Protein Beta 1; DMPK: myotonic dystrophy protein kinase; NGS: next-generation sequencing; APP: Amyloid protein precursor; PSEN1: presenilin-1 PSEN2: presenilin-2; CSF1R: receptor of the colony-stimulating factor-1; DNA: Deoxyribonucleic acid; mtDNA: mitochondrial DNA; HLA-B*51: human leukocyte antigen Β51; SMN1: survival motor neuron 1; CAV3: Caveolin 3; DMD: Duchenne Muscular Dystrophy; CNBP: CCHC-Type Zinc Finger Nucleic Acid Binding Protein; NOTCH3: Neurogenic locus notch homolog protein 3; TTR: Transthyretin; NPC1: Niemann Pick type C protein 1; NPC2: Niemann Pick type C protein 2, ARG1 gene: arginase I gene; EPM2A: epilepsy, progressive myoclonus type 2A; NHLRC1: ncl-1, HT2A and lin-41 repeat-containing protein 1.

## Data Availability

All data generated or analyzed during this study are included in this published article [and its supplementary information files].

## Supplementary Materials

The following supporting information can be downloaded at: https://www.mdpi.com/article/10.3390/medicina59020266/s1, Table S1: Classification of Rare Neurological Diseases diagnosed per disease category.

## Abbreviations

RDs	Rare diseases
RND	Rare Neurological Disease
RNDs	Rare Neurological Diseases
ERN	European Reference Networks
ALS	Amyotrophic lateral sclerosis
MRI	Brain magnetic resonance imaging
NCS	Nerve conduction study
EMG	Electromyography
ECG	Electroencephalography
SPECT	Single-photon emission computed tomography
PET	Positron emission tomography
DAT-Scan	Dopamine transporter scan
TTR	Transthyretin
DNA	Deoxyribonucleic acid
RNA	Ribonucleic acid
Abs	Antibodies
anti-NMDAR	Abs against N-methyl-D-Aspartate Receptor
anti-GAD	Abs against Glutamic acid decarboxylase
PCR	Polymerase chain reaction
RT-QuIC	Real-time quaking-induced conversion
MELAS	Mitochondrial encephalomyopathy, lactic acidosis and stroke-like episodes
hATTR	hereditary TTR-mediated amyloidosis
CNS	Central nervous system
