# Supplementary material for: The Experience of a Tertiary Reference Hospital in the Study of Rare Neurological Diseases
Source: Medicina (Kaunas). 2023 Jan 30;59(2):266. doi: 10.3390/medicina59020266 (PMC9959728; doi:10.3390/medicina59020266)
Supplement: Supplementary file 1 [file medicina-59-00266-s001.zip › medicina-2139224-supplementary.pdf]

Table S1 Classification of Rare Neurological Diseases diagnosed per disease category

DIAGNOSIS	n	%
Neurodegenerative diseases with dementia	***80***	***23***
PPA	29	8.4
FTD	34	9.9
LBD	4	1.1
FTD-MND	2	0.55
Leukodystrohies	2	0.55
RTLA (FTD variant)	4	1.1
CJD	5	1.4
Neurodegenerative diseases with parkinsonism	***11***	***3.2***
CBD	8	2.3
Parkinson plus syndrome	3	0.9
Neurodegenerative diseases with chorea	***9***	***2.6***
Huntington disease	9	2.6
Other rare neurodegenerative diseases	***10***	***3***
MSA	7	2.1
PCA	3	0.9
Rare motor neuron diseases	**64**	**18.7**
ALS - MND	57	16.6
Mills syndrome	1	0.3
MND- Flail arm syndrome	1	0.3
Brown- Vialetto -Van Laere syndrome	1	0.3
SMA	3	0.9
Progressive muscular atrophy	1	0.3
Rare peripheral neuropathies	***30***	***8.8***
CIDP	8	2.3
Guillain Barre syndrome	7	2
Charcot Marie Tooth disease	6	1.8
Isaacs syndrome	3	0.9
MADSAM	2	0.6
hATTR	2	0.6
HNPP	1	0.3
MMN	1	0.3
Rare diseases of the neuromuscular junction	***28***	***8.2***
Myasthenia gravis	27	7.9
Congenital myasthenic syndrome	1	0.3
Genetic skeletal muscle diseases	***19***	***5.6***
Muscular dystrophy	12	3.5
Mitochondrial myopathy	2	0.6
Congenital myotonia	3	0.9

Qualitative or quantitative defects of dystrophin	1	0.3
Henson-Heyck-Landahn myopathy	1	0.3
Rare neuroinflammatory and neuro-immunological disease	***19***	***5.6***
NMOSD	7	2
Autoimmune encephalitis	4	1.1
MOGAD	3	0.9
Paraneoplastic neurologic syndrome	2	0.6
ADEM	1	0.3
PML-IRIS	1	0.3
IgG4-related pachymeningitis	1	0.3
Rare movement disorders	***10***	***3***
PSP	8	2.3
PSP-FTD comlex	1	0.3
Primary orthostatic tremor	1	0.3
Rare systemic or rheumatologic disease with CNS involvement	***11***	***3.2***
Behçet disease	4	1.1
Systemic lupus erythematosus with CNS involvement	3	0.9
Giant cell arteritis	2	0.6
Sarcoidosis	1	0.3
Sjögren syndrome	1	0.3
Rare vascular diseases of the CNS	***8***	***2.3***
CADASIL	4	1.1
Moyamoya disease	2	0.6
Primary angiitis of the central nervous system	1	0.3
Familial cerebral cavernous malformation	1	0.3
Rare ataxias	***7***	***2***
SCA	1	0.3
Friedreich ataxia	3	0.9
Sporadic adult-onset cerebellar ataxia	2	0.6
Paraneoplastic cerebellar degeneration	1	0.3
Rare epilepsies	***5***	***1.5***
Juvenile myoclonic epilepsy	1	0.3
Mesial temporal lobe epilepsy with hippocampal sclerosis	1	0.3
Cerebral malformation with epilepsy	1	0.3
Lafora disease	1	0.3
Inflammatory and autoimmune disease with epilepsy	1	0.3
Neurocutaneous syndrome with epilepsy	***6***	***1.8***

Tuberous sclerosis complex	3	0.9
NF1	2	0.6
Sturge Weber Syndrome	1	0.3
Aqcuired skeletal muscle diseases	***4***	***1.2***
Immune-mediated necrotizing myopathy	2	0.6
Necrotic myopathy unspecified	1	0.3
Polymyositis	1	0.3
Rare inborn errors of metabolism	***4***	***1.2***
MELAS	2	0.6
Pompe disease	1	0.3
Argininemia	1	0.3
CNS malformations	***2***	***0.6***
Congenital hydrocephalus	2	0.6
Rare headache	***2***	***0.6***
Sporadic hemiplegic migraine	1	0.3
Idiopathic intracranial hypotension	1	0,3
Medullar disease	***2***	***0.6***
Transverse myelitis	2	0.6
Rare sleep disorder	***1***	***0.3***
Narcolepsy	1	0.3
Rare nervous system tumor	***1***	***0.3***
Medulloblastoma	1	0.3
Other rare diseases	***9***	***2.6***
Idiopathic intracranial hypertension	3	0.9
Cerebellar ataxia and atrophy	1	0.3
Congenital cerebellar agenesia	1	0.3
Boucher-Naühauser syndrome and cerebellar ataxia	1	0.3
MEN-1 syndrome	1	0.3
Autoimmune autonomic ganglionopathy	1	0.3
Endocardial fibroelastosis - ischemic stroke	1	0.3

PPA: primary progressive aphasias; FTD: frontotemporal dementia; LBD: lewy-body dementia, MND: motor neuron disease; RTLA: right temporal lobe atrophy; CJD: Creutzfeldt-Jakob disease; CBD: corticobasal degeneration; MSA: multiple system atrophy; PCA: posterior cortical atrophy; CNS: Central Nervous System; ALS- MND: amyotrophic lateral sclerosis -motor neuron disease; SMA: spinal muscular atrophy; CIDP: chronic inflammatory demyelinating polyneuropathy; MADSAM: multifocal acquired demyelinating sensory and motor neuropathy; hATTR: hereditary transthyretin amyloidosis; HNPP: hereditary neuropathy with pressure palsies; MMN: multifocal motor neuropathy; NMOSD: neuromyelitis optica spectrum disorder; MOGAD: myelin oligodendrocyte glycoprotein antibody disease; ADEM: acute disseminated encephalomyelitis; PML-IRIS: progressive multifocal leukoencephalopathy - immune reconstitution inflammatory syndrome; PSP: progressive supranuclear palsy; CADASIL: cerebral autosomal dominant arteriopathy with subcortical infarcts and leukoencephalopathy; SCA: spinocerebellar atrophy; NF1: neurofibromatosis type 1; MELAS: mitochondrial encephalomyopathy, lactic acidosis and stroke-like episodes; MEN-1 multiple endocrine neoplasia type 1; n: number of patients
